# Trajectories of depressive symptoms and their predictors in Chinese older population: Growth Mixture model

**DOI:** 10.1186/s12877-023-04048-0

**Published:** 2023-06-16

**Authors:** Yaofei Xie, Mengdi Ma, Wei Wang

**Affiliations:** 1 Department of Psychiatry, Wuhan Mental Health Center, Wuhan, Hubei, China; 2 Department of Psychiatry, Wuhan Hospital for Psychotherapy, Wuhan, Hubei, China; 3 Wuhan Blood Center, Wuhan, Hubei, China; 4 School of Public Health, Xuzhou Medical University, Xuzhou, Jiangsu, China; 5 Center for Medical Statistics and Data Analysis, Xuzhou Medical University, Xuzhou, Jiangsu, China

**Keywords:** Depressive symptoms, Growth mixture model, Older chinese population, Trajectory

## Abstract

**Background:**

Given the rapidly rising proportion of the older population in China and the relatively high prevalence of depressive symptoms among this population, this study aimed to identify the trajectories of depressive symptoms and the factors associated with the trajectory class to gain a better understanding of the long-term course of depressive symptoms in this population.

**Methods:**

Data were obtained from four wave’s survey of the China Health and Retirement Longitudinal Study (CHARLS). A total of 3646 participants who aged 60 years or older during baseline survey, and completed all follow-ups were retained in this study. Depressive symptoms were measured using the 10-item version of the Center for Epidemiologic Studies Depression Scale (CES-D-10). Growth mixture modelling (GMM) was adopted to identify the trajectory classes of depressive symptoms, and both linear and quadratic functions were considered. A multivariate logistic regression model was used to calculate the adjusted odds ratios (ORs) of the associated factors to predict the trajectory class of participants.

**Results:**

A four-class quadratic function model was the best-fitting model for the trajectories of depressive symptoms in the older Chinese population. The four trajectories were labelled as increasing (16.70%), decreasing (12.31%), high and stable (7.30%), and low and stable (63.69%), according to their trends. Except for the low and stable trajectory, the other trajectories were almost above the threshold for depressive symptoms. The multivariate logistic regression model suggested that the trajectories of chronic depressive symptoms could be predicted by being female, living in a village (rural area), having a lower educational level, and having chronic diseases.

**Conclusions:**

This study identified four depressive symptom trajectories in the older Chinese population and analysed the factors associated with the trajectory class. These findings can provide references for prevention and intervention to reduce the chronic course of depressive symptoms in the older Chinese population.

**Supplementary Information:**

The online version contains supplementary material available at 10.1186/s12877-023-04048-0.

## Background

Depression (Major depressive disorders, MDD) in the older population is a public health issue worldwide. According to the World Health Organization (WHO), the older population has a higher prevalence of depression than younger adults, and this number is still growing [1]. Late-life depression can cause the older population to suffer greatly and function poorly within their families, resulting in a significantly lower quality of life and higher mortality [2, 3]. Moreover, in contrast to depression in younger adults, late-life depression is closely related to various chronic diseases, and the comorbidities may lead to more serious health conditions [4].

Some older adults who are affected by depression may not meet the diagnostic criteria of MDD, with subthreshold depression (SD). The prevalence of SD in the older population is reportedly higher than that of MDD. Similar to MDD, SD also has clinical significance due to the impairment in social and occupational functioning [5]. SD can be diagnosed when a core symptom is accompanied by an additional one to three depressive symptoms. Depressive symptoms are a major manifestation of depressive disorders. The occurrence of depressive symptoms in the older population is also highly prevalent and associated with high co-morbidity and increased mortality risk [6–8]. Considering depressive symptoms are easier and more convenient to diagnose than clinical depression, and a considerable number of older patients with MDD are underdiagnosed [9], the measures of depressive symptoms are used more frequently in mental health primary care and other epidemiological investigations to identify people who are at a high risk of depression or are more likely to have MDD or SD.

However, the level of depressive symptoms is not always stable in the long term, and the results of a single measurement may be affected by recent physical conditions or life events. Considering this limitation, longitudinal studies should be adopted to describe the changes in depressive symptoms over time for each individual. This person-centered approach enables a shift in focus from the diagnosis of depressive symptoms to their changes over time. Summarizing the changes into several patterns that show relatively similar developmental trajectories can add to our understanding of the development and premorbid course of depressive symptoms.

Several studies have reported the trajectories of depressive symptoms over time in older populations; for example, Milton et al. [10] identified three patterns of depressive symptom trajectories in the British aged 60 years and older, including non, mild, and moderate severe. By separately modelling the trajectories of depressive symptoms in older women and men aged 65 years and over, Carrière et al. [6] observed two different patterns of trajectories in both sexes, one pattern had a high score on the depressive symptom scale with an increasing trend, while the other pattern had a relatively low score on the scale with a decreasing trend. Three trajectories, including normative, subclinical, and chronic symptoms, have been identified in English older adults aged 65 years and over [11]. Nevertheless, relatively few studies have examined the trajectories for depressive symptoms among the older population, and the existing studies of different populations have also yielded inconsistent results; for example, the number of trajectories identified was different, and trajectories primarily varied in terms of severity (low, medium, and high) and stability (stable, increasing, and decreasing). Detailed results can be found in review by Musliner et al. [12].

To our knowledge, few studies have investigated the changes in depressive symptoms over time in older Chinese population except for a study of older people living in rural China, in which four trajectories were identified, including stably low symptoms, stably high symptoms, increasing symptoms, and decreasing symptoms [13], and a one-year follow-up study among older adults in Hong Kong, which found seven trajectories with different severities of depressive symptoms at baseline (three classes had mild depressive symptoms, two classes were moderate, and two classes were moderately severe) [14]. While the participants of both studies belonged to the Chinese older population, they were not representative of the entire Chinese older population, as one focused on the older population in rural areas and the other was limited to Hong Kong with a one-year follow-up. To address this knowledge gap, this study used seven-year follow-up data from nationally representative samples of the Chinese older population, aiming to analyse the latent growth trajectory and its heterogeneity of depressive symptoms among the Chinese older population, and identify the factors associated with trajectory belonging.

## Materials and methods

### Participants

Data were drawn from four waves surveys of the China Health and Retirement Longitudinal Study (CHARLS) conducted in 2011, 2013, 2015, and 2018. The CHARLS is an ongoing longitudinal study aiming to collect data on the demographic, social, economic, and health statuses of a nationally representative sample of middle-aged and older Chinese residents. A multi-stage stratified probability-proportional-to-size (PPS) sampling method was adopted, and data were collected through face-to-face interviews by trained investigators. The survey data for all waves are publicly available online. In the present study, a total of 4910 participants aged 60 years or over during the baseline survey and completed all subsequent follow-ups were selected. Then those with missing data in the whole depression symptom scale, or those with more than two questions in depressive symptoms scale in any follow-up unanswered or with the responses of “I don’t know” or “I refuse to answer” were removed. After that, a total of 3646 participants were included in the final sample. The proportions of participants removed were presented in **Supplementary Table 1**. There were statistical differences in demographic characteristics including gender, age at baseline, marital status, birthplace, residence, education level, and number of chronic diseases suffered between included and excluded participants.

### Measurement of depressive symptoms

Depressive symptoms were quantified using the 10-item version of the Centre for Epidemiologic Studies Depression Scale (CES-D-10) [15]. The 10 items include three items on depressive affect, five on somatic symptoms, and two on positive affect. Each item has four options; “Rarely or none of the time (< 1 day)”, “Some or a little of the time (1 − 2 days)”, “Occasionally or a moderate amount of the time (3 − 4 days)”, and “Most or all of the time (5 − 7 days)”. The participants were asked to recall and choose the most appropriate frequency of specific feelings and behaviors during the past week. Corresponding to the responses from “rarely or none of the time(< 1 day)” to “most or all of the time (5–7 days)”, each item was rated from zero to three points except “Item 5” and “Item 8”, which were reverse-scored from three to zero. The total score of the CES-D-10 ranged from 0 to 30, and participants who scored ≥ 10 points were considered to have significant depressive symptoms [16]. Cronbach’s alpha of the Chinese version of this scale among the older Chinese population was 0.813 [17].

### Identification of trajectory class of depressive symptoms

Growth mixture modelling (GMM)[18] was used to identify the trajectory class of depressive symptoms. The GMM is a combination of the latent growth curve model (LGCM) and latent class model (LCM), which can simultaneously describe the individual trajectory and distinguish its heterogeneity. It is a type of group-based trajectory modelling that allows for individual variations within each trajectory class. In the present study, four-time CES-D-10 scores were used for GMM analysis to identify growth trajectories and classes of depressive symptoms.

### Predictors of trajectory class

Previous studies have demonstrated that basic demographic characteristics, including sex, age, marital status, educational level, and residence, are associated with depressive symptoms in older Chinese populations [7, 19]. Moreover, physical health status, especially chronic diseases, is closely related to depressive symptoms in older adults [20–22]. Accordingly, we collected demographic information and the number of chronic diseases suffered by the participants to investigate how these variables influenced the latent growth trajectories of depressive symptoms over time in the older Chinese population.

### Statistical analysis

First, we described the distribution of participants and calculated the four-wave CES-D-10 scores for each subgroup. Subsequently, GMM analysis was performed to select the best-fit model, which determined the number of distinct trajectory classes. It contained two steps; the first was to conduct an unconditional model with one class and calculate the model fit indices, including Akaike Information Criterion (AIC), Bayesian Information Criterion (BIC), adjusted Bayesian Information Criterion (aBIC), Entropy and adjusted Lo-Mendell-Rubin likelihood ratio test (aLMR)[23]. Among these indices, lower values of AIC, BIC, and aBIC indicate a better-fitted model, a higher entropy statistic represents higher classification accuracy, and a significant result for aLMR suggests that the model with one more trajectory class fits the data better. The second step was to perform successive analyses by adding a trajectory class each time and comparing the model fit indices across models. Trajectories fitted with both linear and quadratic functions. All GMMs were specified as 200 random sets of starting values to be generated with 10 final stage optimizations. Next, the parameters of the best-fit model and the distribution of the latent growth trajectories of the participants were calculated. Finally, a multivariate logistic regression model was used to calculate the adjusted odds ratios (*OR*s) of associated factors to predict the trajectory of the participants. Descriptive analysis and multivariate logistic regression models were performed using IBM SPSS Statistics version 24, and GMM was established using Mplus 8. All missing values were assumed to be missing at random and were accounted for using regression interpolation. Statistical significance was set at *P* < 0.05, and all *P* values were two-sided.

## Results

### Participants and CES-D-10 scores

Table 1 presents the demographic characteristics and four-wave CES-D-10 scores of the participants. Among the 3646 participants, males and females accounted for approximately 50% each; the average age was 66 years, and nearly 80% were aged 60–69 years old during the baseline survey. Over 80% of the participants were married and approximately three-quarters lived in the village (rural area). Uneducated participants accounted for approximately 30%, and nearly half of the participants completed no further education than the elementary level. Approximately three-quarters of the participants reported that they were diagnosed with at least one type of chronic disease. The average CES-D-10 scores of the four waves were significantly different (*P* < 0.001), among which the score of fourth wave was the highest.

**Table 1:** Participant characteristics by CES-D-10 scores from Wave 1 to Wave 4

Variables	N (%)	CES-D-10 scores (Mean ± SD)			
		Wave 1	Wave 2	Wave 3	Wave 4
Gender					
Male	1834 (50.30)	7.74 ± 5.87	7.15 ± 5.24	7.33 ± 5.97	8.13 ± 6.19
Female	1812 (49.70)	10.07 ± 6.77	9.24 ± 6.20	9.87 ± 6.98	10.61 ± 7.21
**Age at baseline (years old)**					
60–69	2788 (76.47)	8.98 ± 6.45	8.24 ± 5.89	8.68 ± 6.63	9.33 ± 6.83
70–79	791 (21.69)	8.51 ± 6.29	8.01 ± 5.56	8.35 ± 6.54	9.42 ± 6.75
≥ 80	67 (1.84)	9.84 ± 7.61	8.09 ± 6.37	7.87 ± 6.80	9.87 ± 7.62
**Marital status**					
Married	3038 (83.33)	8.62 ± 6.31	7.95 ± 5.66	8.42 ± 6.53	9.15 ± 6.70
Divorced / Widowed / Never married	608 (16.67)	10.29 ± 6.92	9.39 ± 6.48	9.46 ± 6.95	10.42 ± 7.33
**Birthplace**					
Rural	3374 (92.54)	9.05 ± 6.45	8.31 ± 5.85	8.75 ± 6.64	9.54 ± 6.68
Urban	272 (7.46)	6.95 ± 6.02	6.63 ± 5.38	6.65 ± 6.06	7.12 ± 5.94
**Residence**					
Main city zone	440 (12.07)	6.28 ± 5.24	6.27 ± 4.82	6.44 ± 5.92	6.98 ± 5.94
Town	418 (11.46)	7.96 ± 6.04	7.39 ± 5.71	7.62 ± 6.17	8.48 ± 6.48
Village	2788 (76.47)	9.45 ± 6.56	8.61 ± 5.92	9.08 ± 6.70	9.87 ± 6.92
**Education level**					
Illiterate	1114 (30.55)	10.25 ± 6.89	9.21 ± 6.25	10.04 ± 6.89	10.79 ± 7.10
Elementary school or lower	1797 (49.29)	9.02 ± 6.26	8.17 ± 5.69	8.62 ± 6.53	9.45 ± 6.80
Middle school	487 (13.36)	6.93 ± 5.62	6.87 ± 5.14	6.43 ± 5.83	7.18 ± 5.78
High school / Vocational school or greater	248 (6.80)	5.78 ± 5.06	6.27 ± 5.12	6.14 ± 5.51	6.55 ± 5.67
**Number of chronic diseases suffered** ^**†**^					
None	929 (25.48)	6.88 ± 5.82	6.68 ± 5.21	7.02 ± 5.89	7.96 ± 6.40
One	1086 (29.79)	8.23 ± 6.13	7.93 ± 5.66	8.27 ± 6.46	8.84 ± 6.64
Two	796 (21.83)	9.64 ± 6.48	8.67 ± 5.94	8.90 ± 6.59	9.75 ± 6.70
Three or more	835 (22.90)	11.30 ± 6.58	9.73 ± 6.15	10.46 ± 7.12	11.22 ± 7.19
**Total**	3646 (100.00)	8.90 ± 6.44	8.19 ± 5.83	8.59 ± 6.62	9.36 ± 6.83

^†^A total of 14 types of chronic diseases were asked: (1) Hypertension; (2) Dyslipidemia (elevation of low density lipoprotein, triglycerides (TGs), and total cholesterol, or a low high density lipoprotein level); (3) Diabetes or high blood sugar; (4) Cancer or malignant tumor (excluding minor skin cancers); (5) Chronic lung diseases, such as chronic bronchitis, emphysema (excluding tumors, or cancer); (6) Liver disease (except fatty liver, tumors, and cancer); (7) Heart attack, coronary heart disease, angina, congestive heart failure, or other heart problems; (8) Stroke; (9) Kidney disease (except for tumor or cancer); (10) Stomach or other digestive disease (except for tumor or cancer); 11. Emotional, nervous, or psychiatric problems; 12. Memory-related disease; 13. Arthritis or rheumatism; 14. Asthma

### Trajectories of depressive symptoms

Table 2 reports the fit indices of twelve growth mixture models of trajectories of depressive symptoms, including seven linear models (Models 1–7) and five quadratic function models (Models 8–12). First, the linear models were fitted. Three indicators of AIC, BIC, and aBIC prompted to choose the model with a larger number of classes, while the indicator of aLMR suggested that Model 7 was not significantly better than Model 6, and the entropy statistic of Model 6 was also better than Model 7. Therefore, Model 6 was considered to be the best fitting linear model. Further, the quadratic function models were fitted. Also, a larger number of model classes improved the model fit on indicators of AIC, BIC and aBIC. The aLMR statistics indicated that each model was significantly better than the previous one until the number of model classes increased to four. Besides, there was little difference in entropy statistics between Model 11 and Model 12. Consequently, Model 11 was selected to be the best-fit quadratic function model. Finally, the model fit indices of Model 6 and Model 11 were compared. Since the entropy statistics of the two models were equal, the final determination was dependent on the comparison results of AIC, BIC, and aBIC. Thus, the four-class quadratic function model was the best-fit model of the trajectories of depressive symptoms.

**Table 2:** Model fit indices for depressive symptoms across the older Chinese population

Model	LL	AIC	BIC	aBIC	Entropy	aLMR
Linear						
Model 1	-45583.34	91184.68	91240.49	91211.89	-	-
Model 2	-45341.49	90706.98	90781.40	90743.26	0.778	464.808^***^
Model 3	-45263.56	90557.18	90650.14	90602.48	0.772	149.768
Model 4	-45130.41	90296.81	90408.43	90351.24	0.756	255.912^***^
Model 5	-45102.99	90247.97	90378.20	90311.47	0.758	52.696^**^
Model 6	-45062.56	90173.12	90321.95	90245.69	0.762	81.000^**^
Model 7	-45034.64	90123.29	90290.71	90204.92	0.752	63.262
**Quadratic**						
Model 8	-45516.99	91059.98	91140.60	91099.29	-	-
Model 9	-45263.95	90560.95	90666.37	90612.35	0.755	492.032^***^
Model 10	-45126.22	90294.45	90424.67	90357.94	0.777	266.383^*^
**Model 11**	**-45008.01**	**90066.01**	**90221.04**	**90141.60**	**0.762**	**229.443** ^*^
Model 12	-44949.54	89957.08	90136.92	90044.77	0.767	113.470

^*^P < 0.05; ^**^P < 0.01; ^***^P < 0.001

LL = Log likelihood, AIC = Akaike Information Criteria, aBIC = Sample-Size Adjusted Bayesian Information Criteria, aLMR = Lo–Mendell–Rubin Adjusted LRT Test, Bold = Selected model

### Parameters of the chosen model

**Fig. 1:**
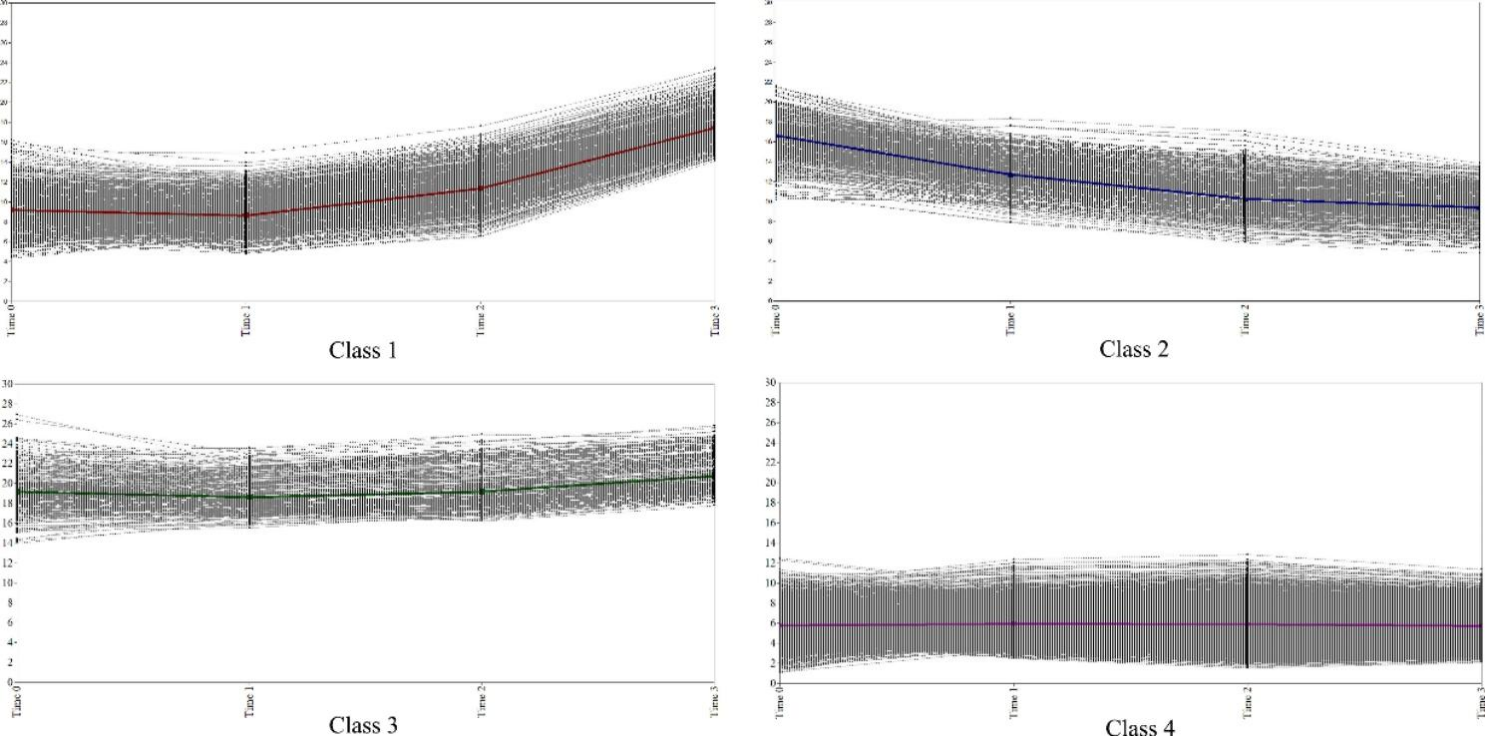
**Trajectories of depressive symptoms in Chinese older population in four-class growth mixture model** (Class 1 could be labelled “increasing”, the estimated mean CES-D-10 scores of this trajectory began at 9.38. The score exceeded the threshold of depressive symptoms in wave 3; Class 2 could be labelled “decreasing”, this group had initially high CES-D-10 scores at 17.97 and decreased to 10.47 at wave 4. Class 3 could be labelled “high and stable” with consistently high CES-D-10 scores; Class 1 could be labelled “low and stable” with consistently low CES-D-10 scores below the threshold of depressive symptoms.)

Table 3 lists the parameters of the quadratic function, slope, and intercept of each trajectory in the four-class quadratic function model. Figure 1 shows the estimated means and individual values of the four trajectories. The trends of the trajectories suggest that the four trajectories could be labelled “increasing”, “decreasing”, “high and stable” and “low and stable”. Among the four trajectories, the “low and stable” trajectory comprised the majority of the participants (63.70%), with an estimated mean CES-D-10 score of 5.79 ± 3.91 at baseline and 5.67 ± 3.74 at the endpoint. The estimated mean CES-D-10 score in “increasing” trajectory (16.68%) at baseline was 9.38 ± 4.66, which was below the threshold of depressive symptoms, and it increased to 11.85 ± 6.41 at wave 3, by which time the CES-D-10 score exceeded the threshold and began to accelerate. The “decreasing” trajectory (12.32%) had an initially high CES-D-10 score of 17.97 ± 4.02 and decreased to 10.47 ± 4.20 at wave 4. The “high and stable” trajectory (7.30%) had the highest initial CES-D-10 score of 19.57 ± 4.55 and stabilized at a high level, with an endpoint of 21.30 ± 4.15 at wave 4.

**Table 3:** Class proportions and parameters of the four-class quadratic model

Classes	N (%)	Quadratic Function	Slope	Intercept
Class 1: Increasing	609 (16.70)	1.660^***^	-2.233^**^	9.196^***^
Class 2: Decreasing	449 (12.31)	0.757^*^	-4.684^***^	16.628^***^
Class 3: High & stable	266 (7.30)	0.532	-1.068	19.164^***^
Class 4: Low & stable	2322 (63.69)	-0.082	0.231	5.771^***^

^*^P < 0.05; ^**^P < 0.01; ^***^P < 0.001

### Predictors of trajectory class

Table 4 presents the results of the multivariate model for predicting the trajectory belonging. Each of the three trajectories of “increasing”; “decreasing”, and “high and stable” was compared to the “low and stable” trajectory. The adjusted *OR*s showed that females were more likely to experience the other three trajectories of depressive symptoms than males. The marital status of divorced, widowed or never married significantly increased the odds of belonging to the “decreasing” trajectory (*OR* = 1.580, 95%CI: 1.211–2.062) and “high and stable” trajectory (*OR* = 1.626, 95%CI: 1.172–2.256). Participants who lived in a town were more likely to be in “decreasing” or “high and stable” trajectories, and living in a village resulted in a higher likelihood of belonging to the other three trajectories. Compared with illiterate participants, those with an educational level of elementary school or lower were less likely to be in the “high and stable” trajectory (*OR* = 0.734, 95%CI: 0.543–0.992). Moreover, middle school and higher educational levels decreased the likelihood of following the other three trajectories. The participants who suffered from one kind of chronic disease were more likely to be in the “high and stable” trajectory, and the more types of chronic diseases suffered, the greater likelihood to be on other trajectories than the “low and stable” trajectory.

**Table 4:** Odds ratios for effects of participant characteristics on trajectory class probabilities^†^

Variables	Odds Ratio (95% CI) (“low and stable” as reference)		
	increasing	decreasing	high and stable
Gender (Male as reference)			
Female	1.731 (1.416–2.117)	1.704 (1.354–2.146)	2.798 (2.051–3.817)
**Age (60–69 years old as reference)**			
70–79 years old	1.143 (0.566–2.310)	0.867 (0.665–1.128)	0.729 (0.514–1.036)
≥ 80 years old	1.036 (0.828–1.297)	1.349 (0.643–2.832)	1.788 (0.777–4.115)
**Marital status (Married as reference)**			
Divorced / Widowed / Never married	1.120 (0.872–1.438)	1.580 (1.211–2.062)	1.626 (1.172–2.256)
**Birthplace (Rural as reference)**			
Urban	0.872 (0.554–1.371)	1.073 (0.639–1.804)	1.331 (0.666–2.660)
**Residence (Main city zone as reference)**			
Town	1.351 (0.879–2.077)	1.850 (1.089–3.143)	3.091 (1.360–7.030)
Village	1.754 (1.211–2.543)	2.639 (1.648–4.226)	5.906 (2.769–12.596)
**Education level (Illiterate as reference)**			
Elementary school or lower	0.889 (0.717–1.102)	0.906 (0.710–1.157)	0.734 (0.543–0.992)
Middle school	0.420 (0.287–0.614)	0.497 (0.326–0.757)	0.576 (0.342–0.969)
High school / Vocational school or greater	0.560 (0.345–0.908)	0.413 (0.219–0.780)	0.413 (0.168–1.013)
**Number of chronic diseases suffered** **(None as reference)**			
One	1.253 (0.974–1.612)	1.279 (0.935–1.749)	1.826 (1.177–2.835)
Two	1.606 (1.230–2.097)	2.003 (1.458–2.752)	2.538 (1.616–3.986)
Three or more	1.964 (1.500–2.570)	3.498 (2.573–4.732)	6.164 (4.056–9.368)

^**†**^ The *OR*s were adjusted for gender, marriage status, residence, education level, number of chronic diseases suffered

OR: odds ratio and CI: confidence intervals

## Discussion

China has the largest older population worldwide, and its aging population continues to intensify. Moreover, according to national survey reports, the prevalence of depressive symptoms among the older Chinese population was high [8]. This study aimed to explore how depressive symptoms evolve in the older Chinese population and identify the predictors of trajectory belonging. These findings are expected to provide a reference for the prevention and early intervention of depressive symptoms in older adults.

### Trajectories of depressive symptoms

Based on the longitudinal data of the seven-year follow-up study, we revealed four trajectories of depressive symptoms in older Chinese populations; including low and stable, increasing, decreasing, and high and stable, accounting for 63.69%, 16.70%, 12.31%, and 7.30%, respectively. These heterogeneous patterns were consistent with the findings of Xiang [24], Lin et al. [25] and Kuchibhatla et al. [26], in which four similar trajectories were identified in the older population, with a majority of participants in the low and stable group and less than 10% in the high and stable groups. It is worth noting that in the study by Kuchibhatla et al., the trajectories of both increasing and decreasing groups were below the threshold of positive depressive symptoms, while in the present study, participants in the increasing group had relatively higher initial CES-D-10 scores, which were close to the threshold of depressive symptoms. The estimated scores of the decreasing group were also consistently above the threshold of depressive symptoms, which indicated that although the degree of depressive symptoms showed a decreasing trend, it failed to relieve to a negative level of depressive symptoms. Thus, the four trajectories represented four classes: stable non-onset, onset after gradual deterioration, improvement but not recovery, and severe chronic conditions.

Other studies have reported four trajectory classes, but the trajectory patterns were not the same as our findings; for example, in the study by Hsu et al.[27], instead of a high and stable group, there was a medium group, and in the study by Kuo et al.[28], there was a persistent mild group that replaced the decreasing group. In addition, some studies revealed different numbers of trajectory classes (e.g. three [10, 29], five [30, 31], six [32, 33]). The variation among these research results may be caused by different demographics of the population, follow-up intervals, or differences in the measurement tools of depressive symptoms. In addition, participants from different countries or regions may have different residential environments, which may result in differences in the trajectory patterns of depressive symptoms. Ruiz et al. [34] reported that older English populations with different levels of perceived neighborhood social cohesion had different depressive symptom trajectory patterns. Petkus et al. [35] indicated that long-term exposure to particulate matter with aerodynamic diameter of ≤ 2.5 mm (PM2.5) was associated with depression trajectories among older women.

### Predictors of trajectory class

Given that depressive symptoms progress differently among individuals, the exploration of predictors for trajectory classes can provide information on how to stratify older populations. Our findings suggest that all increasing, decreasing, high and stable depressive trajectories could be predicted by being female, living in a village, having a lower education level, and having comorbidity of chronic diseases. Furthermore, the decreasing, high and stable trajectories could also be predicted by the marital status of the divorced, widowed, or never married. These results are consistent with the general conclusion of previous studies that the explored factors associated with depressive symptoms in older population [36–38]. In addition, it is noteworthy that the differences in the CES-D-10 scores of the four trajectories had already appeared at baseline. Therefore, it can be inferred that participants in different trajectories might have experienced varying degrees of harmful exposure before the baseline survey and that these exposures, which may still have an impact on the development of depressive symptoms, may not have been measured or included in the present study.

### Limitations

The present study has several limitations. First, although the depressive symptoms of the same individual were repeatedly measured, the follow-up interval was long; therefore, the between-wave transitory changes in depressive symptoms could not be monitored. Second, although the sample size was sufficient to obtain reliable model estimates, a considerable number of older adults were excluded from the study because they failed to complete all follow-ups. Loss follow-up may be associated with the deterioration of depressive symptoms, which may affect the model estimation. In addition, part of participants were excluded because they missed too much data on the depressive symptom scale. There were statistical differences in the proportions of included and excluded participants. Although significant statistical inferences are more likely to be obtained when the sample size is large, the statistical differences between included and excluded participants should be fully considered when interpreting the results. Third, only a limited number of factors were included in the predictor analysis, and the associations between these factors and depressive trajectory classes might be partly due to their associations with baseline depressive levels.

## Conclusion

This is the first study to explore the seven-year trajectories of depressive symptoms in an older Chinese population. Four depressive symptom trajectories—low and stable, increasing, decreasing, and high and stable, were identified using growth mixture modelling. Except for the low and stable trajectory, which showed a persistently low level of depressive symptoms, the other trajectories were almost above the threshold of depressive symptoms, including the decreasing trajectory. The significant demographic predictors of these trajectories were sex, marital status, residence, and educational level. In addition, having chronic diseases was associated with more severe depressive symptoms. Our results demonstrate that depressive symptoms are difficult to relieve naturally once they occur in the Chinese older population, therefore, screening for depressive symptoms could be carried out in routine physical examinations to identify older population with positive depressive symptoms, and early intervention efforts, such as family and community support and psychosocial intervention by primary health care workers, can be undertaken in specific populations to avoid long-term exposure to depressive symptoms, which can lead to an increased risk of clinical depression. Regular follow-ups are also advocated for positive individuals, therefore, interventions could be tailored to target specific needs for each symptom trajectory.

## Electronic supplementary material

Below is the link to the electronic supplementary material.

Supplementary Material 1

## Data Availability

The datasets analyzed during the current study are available in the CHARLS repository: http://charls.pku.edu.cn/index/en.html.

## **Abbreviations**

AIC: Akaike information criterion
aBIC: Adjusted Bayesian information criterion
aLMR: Adjusted Lo-Mendell-Rubin likelihood ratio test
BIC: Bayesian information criterion
CHARLS: China Health and Retirement Longitudinal Study
CES-D-10: The 10-item version of the Centre for Epidemiologic Studies Depression Scale
GMM: Growth mixture model
LCM: Latent class model
LGCM: Latent growth curve model
MDD: Major depressive disorder
OR: Odds ratio
PM2.5: Particulate matter with aerodynamic diameter of ≤ 2.5 mm
SD: Subthreshold depression
PPS: Probability-proportional-to-size
WHO: World Health Organization
95% CI: The 95% confidence intervals
