# Supplementary material for: Trajectories of depressive symptoms and their predictors in Chinese older population: Growth Mixture model
Source: BMC Geriatr. 2023 Jun 16;23:372. doi: 10.1186/s12877-023-04048-0 (PMC10276362; doi:10.1186/s12877-023-04048-0)
Supplement: Supplementary file 1 — Supplementary Material 1 [file 12877_2023_4048_MOESM1_ESM.docx]

**Supplementary Table 1 Characteristics of included and excluded participants**

| **Variables** | **Included**  **N (%)** | **Excluded** | ***χ*^2^** |
| --- | --- | --- | --- |
|  |  | **N (%)** |  |
| **Gender** |  |  | 4.933^*^ |
| Male | 1834 (50.30) | 590 (46.68) |  |
| Female | 1812 (49.70) | 674 (53.32) |  |
| **Age at baseline (years old)** |  |  | 399.849^***^ |
| 60-69 | 2788 (76.47) | 644 (50.95) |  |
| 70-79 | 791 (21.69) | 463 (36.63) |  |
| ≥80 | 67 (1.84) | 157 (12.42) |  |
| **Marital status** |  |  | 32.375^***^ |
| Married | 3038 (83.33) | 962 (76.11) |  |
| Divorced / Widowed / Never married | 608 (16.67) | 302 (23.89) |  |
| **Birthplace** |  |  | 8.424^**^ |
| Rural | 3374 (92.54) | 1199 (94.86) |  |
| Urban | 272 (7.46) | 65 (5.14) |  |
| **Residence** |  |  | 11.358^**^ |
| Main city zone | 440 (12.07) | 136 (10.76) |  |
| Town | 418 (11.46) | 122 (9.65) |  |
| Village | 2788 (76.47) | 1006 (79.59) |  |
| **Education level** |  |  | 204.379^***^ |
| Illiterate | 1114 (30.55) | 449 (35.52) |  |
| Elementary school or lower | 1797 (49.29) | 667 (52.77) |  |
| Middle school | 487 (13.36) | 102 (8.07) |  |
| High school / Vocational school or greater | 248 (6.80) | 46 (3.64) |  |
| **Number of chronic diseases suffered^†^** |  |  | 9.294^*^ |
| None | 929 (25.48) | 373 (29.51) |  |
| One | 1086 (29.79) | 362 (28.64) |  |
| Two | 796 (21.83) | 243 (19.22) |  |
| Three or more | 835 (22.90) | 286 (22.63) |  |
| **Total** | 3646 (100.00) | 1264 (100.00) |  |

^*^*P* < 0.05; ^**^*P* < 0.01; ^***^*P* < 0.001
